# Glycyrrhizin protects against sodium iodate‐induced RPE and retinal injury though activation of AKT and Nrf2/HO‐1 pathway

**DOI:** 10.1111/jcmm.14246

**Published:** 2019-03-01

**Authors:** Huijun He, Daheng Wei, Hua Liu, Chen Zhu, Yue Lu, Zongwen Ke, Shuang Jiang, Jianhua Huang

**Affiliations:** ^1^ First Affiliated Hospital of Jinzhou Medical University Jinzhou China; ^2^ Graduated School of Jinzhou Medical University Jinzhou China; ^3^ Third Affiliated Hospital of Jinzhou Medical University Jinzhou China; ^4^ Life Science Institute of Jinzhou Medical University Jinzhou China

**Keywords:** glycyrrhizin, Nrf2/HO‐1 pathway, retinal injury AKT pathway, retinal pigment epithelium

## Abstract

Glycyrrhizin is a bioactive triterpenoid saponin extracted from a traditional Chinese medicinal herb, glycyrrhiza, and has been reported to protect the organs such as liver and heart from injuries. However, there is no report about the effects of glycyrrhizin on atrophic age‐related macular degeneration (AMD). This study investigated the effects of glycyrrhizin on retinal pigment epithelium (RPE) in vitro and retina of mice in vivo treated with sodium iodate (SI). Glycyrrhizin significantly inhibited SI‐induced reactive oxygen species (ROS), and decreased apoptosis of RPE in vitro. The underlying mechanisms included increased phosphorylation of Akt, and increased expression of nuclear factor erythroid 2‐related factor2 (Nrf‐2) and HO‐1, thereby protecting RPE from SI‐induced ROS and apoptosis. Furthermore, glycyrrhizin significantly decreased the apoptosis of retinal cells in vivo, resulting in the inhibition of thinning of retina, decreasing the number of drusen and improving the function of retina. These findings suggested that glycyrrhizin may be a potential candidate for the treatment of atrophic AMD in clinical practice.

## 1 INTRODUCTION

Irreversible blindness is most commonly seen in individuals aged over 65, and one of the major causes for this is age‐related macular degeneration (AMD).^1^ It is divided into ‘dry’ and ‘wet’ types.^2^, ^3^, ^4^, ^5^ The wet form of AMD triggers abnormal angiogenesis underneath the retina, leading to rapid vision loss. In the dry form of AMD, the retinal cells die progressively, displaying geographic atrophy. Thus, the ‘dry’ AMD was also known as atrophic AMD, accounting for about 10%–20% of cases of legal blindness from AMD. While the exact etiology of atrophic AMD is not known, and a number of risk factors, such as old age, genetic susceptibility and inflammation are suggested to act on the outer retina (RPE and photoreceptors) to disrupt normal cellular homeostasis, resulting in the occurrence of the disease.^6^ Generally, when normal cells are exposed to stress conditions, the compensatory pathways in the cells were initially activated to maintain homeostasis; however, in chronic or intense stress conditions, apoptosis occurs through various signalling pathways. A major objective for treating atrophic AMD involves reduction in retinal pigment epithelium (RPE) and photoreceptor death induced by various pathologic factors. Despite substantial progress in the development of new therapies for wet AMD using inhibition of vascular endothelial growth factor (VEGF), the severe visual impairment associated with geographic atrophy in dry AMD remains untreatable.^7^ Recently, medicinal plants that produce ursolic acid have been reported to successfully prevent cell death in heart, liver and neurosystem.^8^ Thus, it is necessary to investigate natural compounds derived from plants for the prevention of atrophic AMD.

Glycyrrhizin, also known as glycyrrhizic acid, is a substance extracted from a traditional Chinese medicinal herb, Glycyrrhiza. According to a previous report, glycyrrhizin reduced inflammatory injury in a model of inflammatory pain via suppression of NF‐κB, TNF‐α and intercellular adhesion molecules.^9^ Besides anti‐inflammatory, glycyrrhizin has also been reported to have anti‐viral and hepatoprotective effects, and was given to patients with chronic hepatitis and human immunodeficiency virus infection.^10^, ^11^ Furthermore, glycyrrhizin possessed potential neuroprotective effect against ischaemia‐reperfusion injury in a model of focal cerebral ischaemic‐reperfusion injury.^12^ However, whether glycyrrhizin prevents atrophic AMD is still unknown.

Sodium iodate increases reactive oxygen species (ROS) and induces consistent and selective damage to the RPE.^13^ Exposure to sodium iodate (SI) results in a primary death of the RPE followed by a secondary death of the overlying photoreceptors, similar to what is observed in advanced atrophic AMD. Thus, SI has been widely used to study the molecular mechanism of cell death in atrophic AMD.^14^, ^15^, ^16^, ^17^ Hence, in this study, we investigated whether glycyrrhizin prevents SI‐mediated RPE injury in vitro and retinal injury in vivo. Glycyrrhizin prevented SI‐induced RPE and retinal injury through inhibition of ROS and decreased apoptosis, indicating a potential clinical usage of glycyrrhizin in protecting against atrophic AMD.

## 2 MATERIALS AND METHODS

### 2.1 Reagents and cell line

Glycyrrhizin was purchased from the National Institute for Food and Drug Control (Nanjing, China). Sodium iodate was procured from Sigma Industrial Company (Shanghai, China, purity 98%). Anti‐p‐Akt (Ser473), anti‐Akt, anti‐Nrf2, anti‐HO‐1, anti‐cleaved caspase‐3 and anti‐β‐actin were obtained from Cell Signaling Technology (Shanghai, China). Human adult pigment epithelial‐19 (ARPE‐19) was purchased from American Type Culture Collection (ATCC, Manassas, VA, USA).

### 2.2 Cell viability assay

Glycyrrhizin was dissolved in 0.1% dimethyl sulfoxide (DMSO). The human ARPE‐19 cells were divided into three groups: control group (DMSO), sodium group (SI) and sodium group plus glycyrrhizin (SI + GA). Human ARPE‐19 cells were plated at a density of 1 × 10^4^ cells/well in complete medium containing 10% fetal bovine serum (FBS) in 96‐well plates. After cell attachment, the cells were pre‐treated with a series of concentrations of glycyrrhizin (25‐200 μmol) for 6 hours before adding SI (1200 μg/mL). After another 24 hours of cell culture, 20 μL of methylthiazolyldiphenyl‐tetrazolium bromide (MTT) reagent was added to each well, and incubated for 4 hours at 37°C. After that, the cell culture medium was carefully aspirated, and 150 μL of DMSO was added to each well. The 96‐well plates were shaken at room temperature for 10 minutes, and then the absorbance was measured on a microplate reader at 570 nm. Wells without cells were used as blank controls.

### 2.3 Cell apoptosis assay

After pre‐treatment of ARPE‐19 cells for 6 hours with glycyrrhizin (50 μmol) in six‐well plate, SI was added to the cell culture. After 24 hours of cell culture, hochest 33342 staining was performed, and apoptotic cells were identified based on the morphological changes in the nuclear assembly involving chromatin condensation and fragmentation. Annexin V and propidium iodide staining were also used to detect apoptotic cells by flow cytometry. Briefly, after 6 hours pre‐treatment of ARPE‐19 cells with 50 μmol of glycyrrhizin, SI was added to the cell culture. After 24 hours of cell culture, ARPE‐19 cells were digested with 0.25% trypsin and then washed with phosphate buffered saline (PBS). Cells were collected by centrifugation at 280 *g* for 5 minutes, followed by addition of 500 μL binding buffer, 5 μL Annexin V‐FITC, and 5 μL PI. Subsequently, the samples were incubated at room temperature in the dark for 10 minutes, followed by flow cytometry within 1 hour. The percentages of early and late apoptotic cells were calculated.

### 2.4 Measurement of ROS production

Dichloro‐dihydro‐fluorescein diacetate (DCFH‐DA) method was used to assess the ROS levels in RPE cells. ARPE‐19 cells were pre‐treated with glycyrrhizin (50 μmol) for 6 hours before the addition of SI to the cell culture. After 24 hours of cell culture, the ARPE‐19 cells were incubated with 2,7‐dichlorofluorescin diacetate (20 μmoL) (ROS assay kit, E004; Nanjing Jiancheng Bioengineering Corporation, Nanjing, China) at 37°C for 60 minutes in the dark. After that, the ARPE‐cells were observed under an Olympus IX71 fluorescence microscope (Tokyo, Japan). Five high power fields were randomly selected. Fluorescence intensities of the sections stained for ROS were determined with the Image‐Pro Plus software.

### 2.5 Animals

All animal experiments were approved by the Experimental Animal Ethics Committee of Jinzhou Medical University and conformed to the Guide for the Care and Use of Laboratory Animals published by the USA National Institutes of Health (Publication, 8th Edition, 2011). Healthy male C57BL/6 mice (weight 20‐25 g) were purchased from Beijing Vital River Laboratory Animal Technology Co., Ltd., Beijing, China. The mice were allowed free access to standard chow and water at room temperature 22‐24°C and 12 hour light/dark cycle. The animals were acclimatized for a minimum of 1 week before the experiments.

### 2.6 Animal experimental setting

Thirty mice were randomly divided into three groups: sham (n = 10), SI (n = 10), and SI + glycyrrhizin treatment (n = 10). In the sham group, 0.1% DMSO in double distilled water (DDW) was injected intraperitoneally daily for 1 week before the mice were intravenously injected with DDW containing no SI one time, and intraperitoneal administration of 0.1% DMSO in DDW was continued until the mice were sacrificed. In the SI group, 0.1% DMSO in DDW was injected intraperitoneally daily for 1 week before the mice were intravenously injected with SI in DDW (30 mg/kg) one time, and intraperitoneal administration of 0.1% DMSO in DDW was continued until the mice were sacrificed. In the SI + glycyrrhizin group, glycyrrhizin in 0.1% DMSO (50 mg/kg/d) was injected intraperitoneally daily for 1 week before the mice were intravenously injected with SI in DDW (30 mg/kg) one time, and intraperitoneal administration of glycyrrhizin in 0.1% DMSO was continued until the mice were sacrificed.

### 2.7 Micron IV imaging

Mice were anaesthetized by intraperitoneal injection of ketamine (100 mg/kg body weight) and xylazine (10 mg/kg body weight). Pupils were dilated with topical administration of 2.5% phenylephrine containing 0.5% tropicamide, and the cornea was anaesthetized with 0.5% proparacaine. The retinas of the mice were imaged in vivo using the Micron IV retinal imaging camera system (Phoenix Research Laboratories, Pleasanton, CA). Optical coherence tomography was performed on days 0 and 28 to monitor the retinal morphology.

### 2.8 Electroretinography

Mice were dark adapted overnight and anaesthetized by intraperitoneal injection of ketamine (100 mg/kg body weight) and xylazine (10 mg/kg body weight). Pupils were dilated with topical administration of 2.5% phenylephrine containing 0.5% tropicamide, and the cornea was anaesthetized with 0.5% proparacaine. Full‐field electroretinography (ERG) was measured using a non‐attenuated light stimulus. Photoreceptor responses (a‐wave) and bipolar cells responses (b‐wave) were elicited with a flash (5.6 cds/m^2^), and recorded within a period of 5 seconds after the flash presentation signals were amplified and averaged on a PC‐based recording system. The a‐wave amplitude was measured from the baseline to the trough of the a‐wave, while the b‐wave amplitude was measured from the trough of the a‐wave to the peak of the b‐wave.

### 2.9 Haematoxylin and eosin staining

Five mice in each group were sacrificed on day 28 after SI treatment, and eye samples were harvested, embedded in paraffin, and sliced into 4‐μm‐thick sections. Subsequently, the sections were stained with haematoxylin and eosin (HE). Images of retina at the same position in each section of the individual eye were selected and photographed under microscope.

### 2.10 Terminal dUTP nick end‐labelling** assay**

In situ cell death detection kit (Roche, Indianapolis, IN) was used to detect apoptosis in the retina of mice according to the manufacture's protocol. The samples were washed with PBS three times, and then were fixed in 4% paraformaldehyde for 1 hour and permeabilized in 0.1% Triton X‐100 sodium citrate buffer for 2 minutes. The sections were then randomly selected for terminal dUTP nick end‐labelling (TUNEL), and the apoptotic cells were revealed using the diaminobenzidine (DAB) kit. Nuclei were counterstained with haematoxylin. The total number of TUNEL‐positive nuclei was enumerated in five randomly chosen fields of view per tissue section in a blinded manner, and the results were expressed as the ratio of TUNEL‐positive nuclei to total nuclei/field.

### 2.11 Western blot

Retinal pigment epithelium samples were collected and lysed with lysis buffer containing 20 mmol/L 4‐(2‐Hydroxyethyl)piperazine‐1‐ethane‐sulfonic acid (HEPES) (pH 7.5), 150 mmol/L NaCl, 1 mmol/L ethylenediaminetetraacetic acid, 0.5% Triton X‐100 and protease inhibitors (Roche). An equivalent of 40 μg total protein extract was separated by SDS‐PAGE, transferred onto nitrocellulose membranes and blocked for 1 hour at room temperature. For detecting protein expression, the membranes were probed overnight at 4°C with primary antibodies (dilution 1:1000), followed by anti‐mouse or anti‐rabbit secondary antibodies (dilution 1:2000) conjugated to horseradish peroxidase (Zymed Inc, San Francisco, CA) for 1 hour at room temperature. The enhanced chemiluminescence ECL Plus system (Amersham Biosciences, Little Chalfont, UK) was used to evaluate the immunoreactivity. β‐actin (1:1000) was used as an internal control. The intensity of the bands was measured with a scanning densitometer (Bio‐Rad, Shanghai, China) coupled with Bio‐Rad analysis software.

### 2.12 Statistical analysis

All the values were presented as mean ± SD. One‐way ANOVA followed by Bonferroni/Dunn test were performed for group comparisons. A *P*‐value of <0.05 was considered statistically significant.

## 3 RESULTS

### 3.1 Glycyrrhizin decreased the apoptosis of RPE treated with SI in vitro

**Figure 1:**
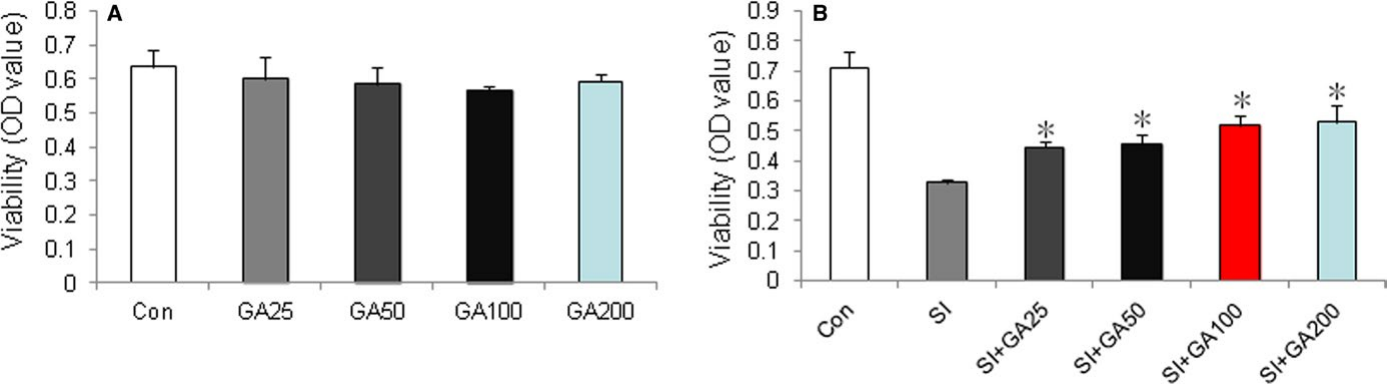
Effects of glycyrrhizin on viability of ARPE‐19 cells in vitro. A, methylthiazolyldiphenyl‐tetrazolium bromide (MTT) assay showed that glycyrrhizin had no toxic effects on ARPE‐19 cells at concentrations of 25‐200 μmol. B, MTT assay showed that glycyrrhizin at a concentration of 25‐100 μmol significantly increased the viability of ARPE‐19 cells treated with sodium iodate, compared with sodium iodate control (*P < 0.01).

**Figure 2:**
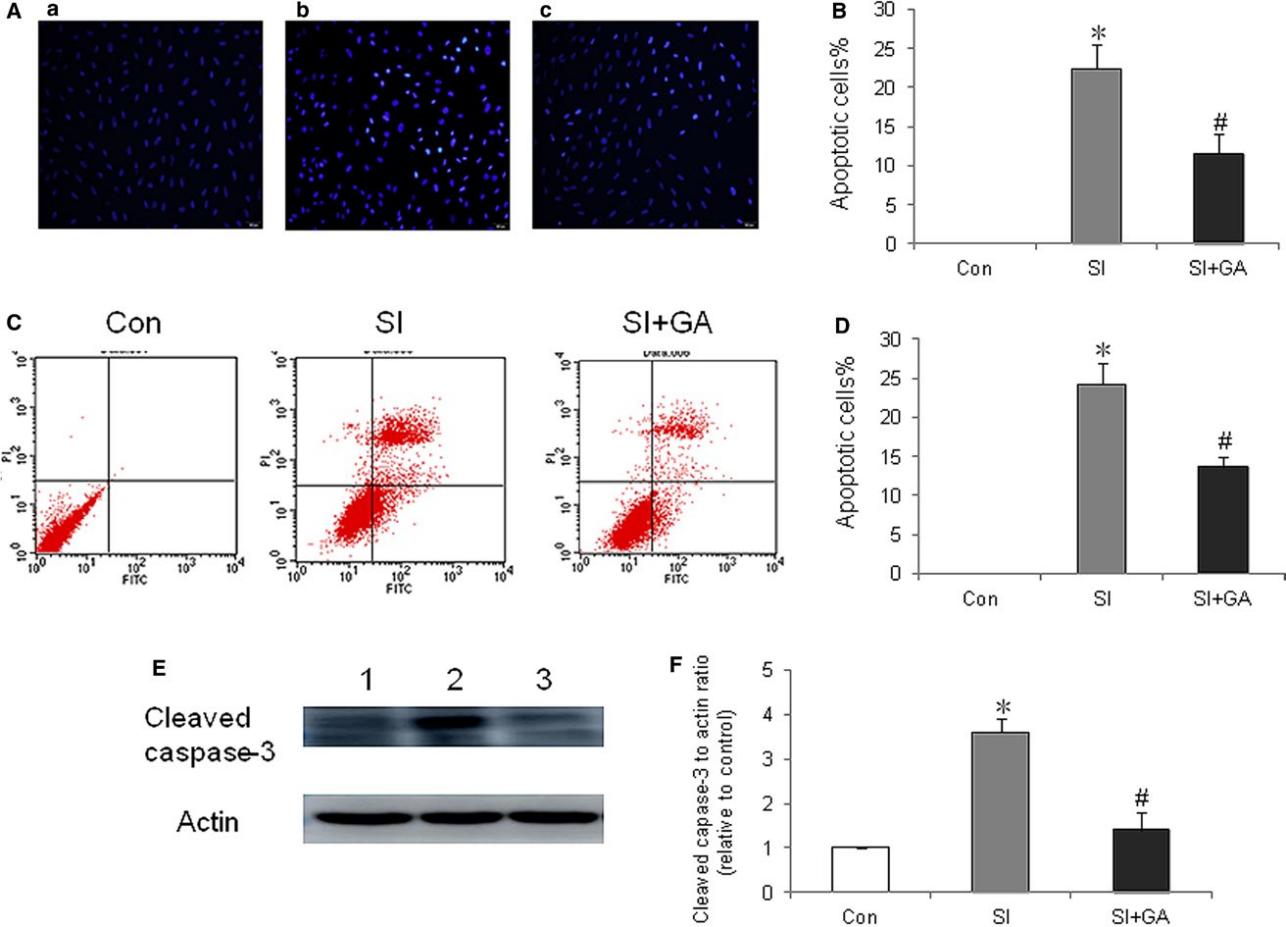
Effects of glycyrrhizin on apoptosis of ARPE‐19 cells in vitro. A, Hochest 33342 staining of ARPE‐19 cells were treated with sodium iodate at a concentration of 50 μmol. a, sham; b, retinal pigment epithelium (RPE) treated with sodium iodate (SI); c, RPE treated with sodium iodate plus glycyrrhizin (SI + GA). B, Sodium iodate significantly increased apoptosis of ARPE‐19 cells, compared normal control (*P < 0.01), while glycyrrhizin significantly inhibited apoptosis of ARPE‐19 cells treated with sodium iodate, compared with sodium iodate control (^#^ *P < *0.01). C, Flow cytometry analysis of apoptosis of ARPE‐19 cells treated with SI at a concentration of 50 μmol. D, Sodium iodate significantly increased apoptosis of ARPE‐19 cells compared normal control (*P < 0.01), while glycyrrhizin treatment significantly decreased the apoptosis of ARPE‐19 cells treated with SI, compared with SI control (^#^ *P < *0.01). E, Western blot detection of the effect of glycyrrhizin on cleaved caspase‐3 in RPE treated with sodium iodate. Lane 1, sham; Lane 2, RPE treated with SI; Lane 3, RPE treated with SI + GA. F, Sodium iodate significantly increased expression of cleaved capase‐3 in ARPE‐19 cells compared with normal control (*P < 0.01), while glycyrrhizin significantly decreased the expression of cleaved‐caspase 3 in ARPE‐19 cells treated with sodium iodate, compared with the sodium iodate control (^#^ *P < *0.01).

Methylthiazolyldiphenyl‐tetrazolium bromide assay was used to evaluate the effect of glycyrrhizin on the viability of ARPE‐19 cells treated with SI. The results showed that glycyrrhizin at various concentrations of 25‐100 μmol showed no toxicity on ARPE‐19 cells and significantly increased its viability, compared with the SI control (Figure 1A,B). Hochest 33342 staining showed that the apoptotic nuclei of ARPE‐19 cells were overtly reduced at 50 μmol of glycyrrhizin after SI treatment (Figure 2A). Flow cytometry showed that glycyrrhizin at 50 μmol significantly inhibited ARPE‐19 cells apoptosis after SI treatment (Figure 2B). Western blot also showed that glycyrrhizin at 50 μmol significantly inhibited the expression of cleaved‐Capase‐3 in ARPE‐19 cells treated with SI (Figure 2C).

### 3.2 Glycyrrhizin decreased ROS in RPE treated with SI in vitro

**Figure 3:**
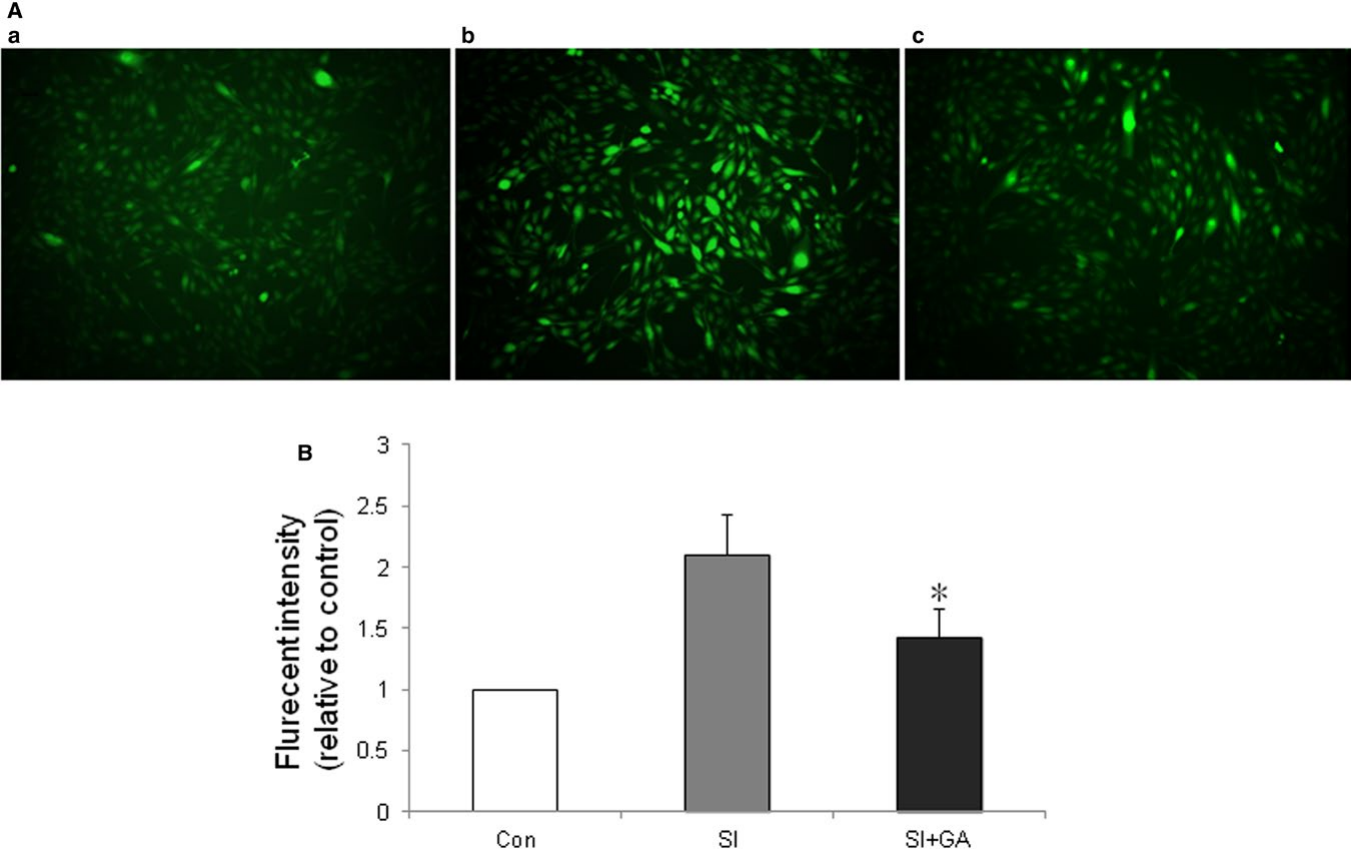
Effects of glycyrrhizin on reactive oxygen species (ROS) in ARPE‐19 cells treated with sodium iodate in vitro*. *Dichloro‐dihydro‐fluorescein diacetate (DCFH‐DA) staining of ARPE‐19 cells after 24 h of sodium iodate treatment. A, sham; b, retinal pigment epithelium (RPE) treated with sodium iodate (SI); c, RPE treated with sodium iodate plus glycyrrhizin. B, Glycyrrhizin (50 μmol) significantly decreased ROS levels in ARPE‐19 cells after treatment of sodium, compared with sodium iodate control (*P < 0.01).

The DCFH‐DA method was used to evaluate the effects of glycyrrhizin on ROS production in RPE after treatment with SI. The results showed that glycyrrhizin at 50 μmol significantly decreased the ROS levels in the ARPE‐19 cells after SI treatment, compared with SI control (Figure 3A,B).

### 3.3 Glycyrrhizin decreased apoptosis of retina induced by SI in vivo

**Figure 4:**
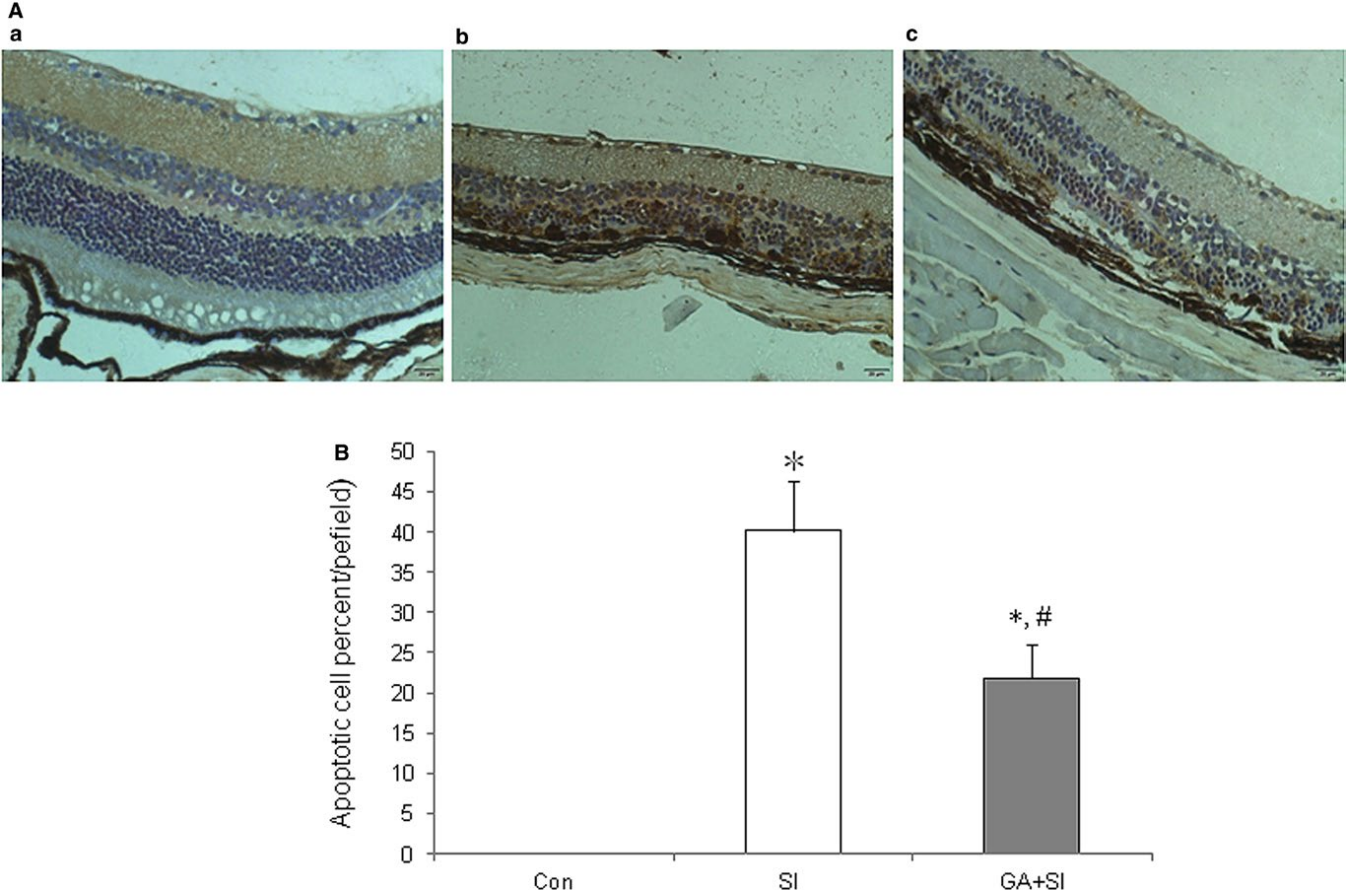
Effects of glycyrrhizin on retinal apoptosis after sodium iodate (SI) treatment in vivo. A, Terminal dUTP nick end‐labelling staining of retina after 28 d of SI treatment. a, sham; b, retina treated with SI; c, retina treated with SI plus glycyrrhizin (SI + GA). Apoptotic cell nuclei were stained brown. B, Both SI and SI + GA significantly increased apoptosis of retina, compared with normal control (*P < 0.01), however, glycyrrhizin significantly decreased the apoptosis of retina treated with SI, compared with SI control (^#^ *P* < 0.01)

Terminal dUTP nick end‐labelling staining was used to detect the effects of glycyrrhizin on apoptosis of retina on day 28 after treatment of SI in vivo. The results showed that glycyrrhizin significantly decreased apoptosis of retina treated with SI (Figure 4A,B).

### 3.4 Glycyrrhizin decreased thinning of retina and number of drusen induced by SI

**Figure 5:**
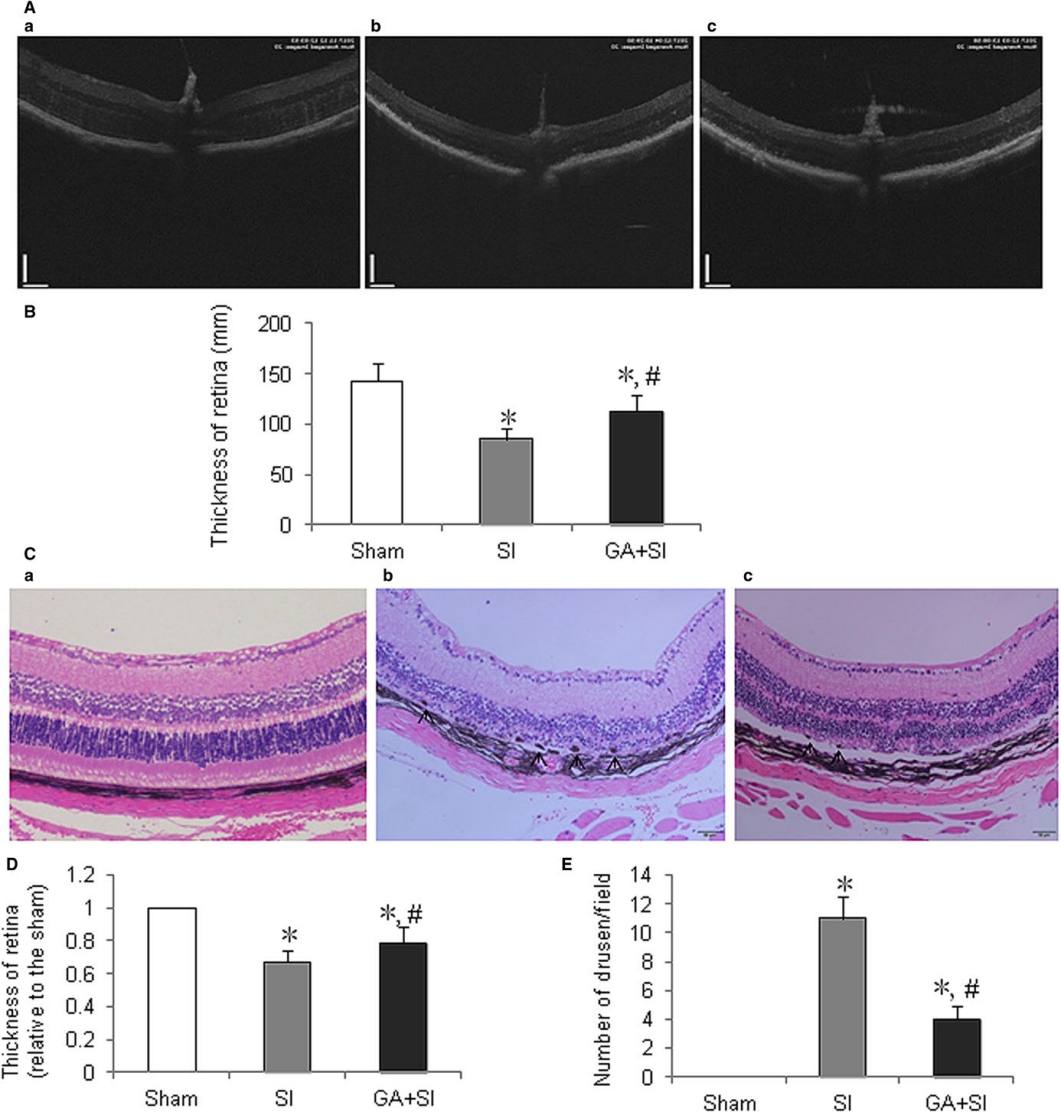
Effects of glycyrrhizin on the thinning of retina and drusen number of retina in vivo. A, Optical coherence tomography measurement of the effects of glycyrrhizin on retina treated with sodium iodate (SI) in mice. a, Sham; b, retina treated with SI; c, retina treated with SI plus glycyrrhizin (SI + GA). B, Both SI and SI + GA significantly increased thinning of retina, compared with normal control (*P < 0.01), however, glycyrrhizin significantly inhibited thinning of retina after treatment of SI on day 28, compared with SI control (^#^ *P* < 0.05). C, Haematoxylin and eosin staining of retina on day 28 after SI treatment. a, sham; b, retina treated with SI; c, retina treated with SI + GA. Both SI and iodate and sodium plus GA had disorganization of Bruch's membrane, however, glycyrrhizin overtly alleviated the disorganization of Bruch's membrane caused by SI. D, Both SI and SI + GA significantly increased thinning of retina, compared normal control (*P < 0.01), however, glycyrrhizin significantly inhibited thinning of retina after treatment of sodium, compared with SI control (^#^ *P < *0.05). E, Both SI and SI + GA significantly increased the drusen number of retina, compared with normal control (*P < 0.01), however, glycyrrhizin significantly decreased the drusen number of retina after treatment of sodium, compared with SI control (^#^ *P* < 0.01)

Optical coherence tomography was performed to detect the effects of glycyrrhizin on the injury of retina induced by SI. The results showed that glycyrrhizin, at the end of the detection points, significantly decreased thinning of retina caused by SI treatment, compared with SI control (Figure 5A,B).

To further prove this, haematoxylin and eosin staining was performed to detect the effects of glycyrrhizin on the pathological changes of retina after SI administration. The results showed that at the end of detection points, glycyrrhizin significantly decreased the thinning of retina, especially outer nuclear layer (ONL) and inner nuclear layer (INL) (Figure 5C,D). The data also showed that the drusen number was significantly decreased by glycyrrhizin (Figure 5C,E).

### 3.5 Glycyrrhizin increased ERG amplitudes in SI‐treated mice

**Figure 6:**
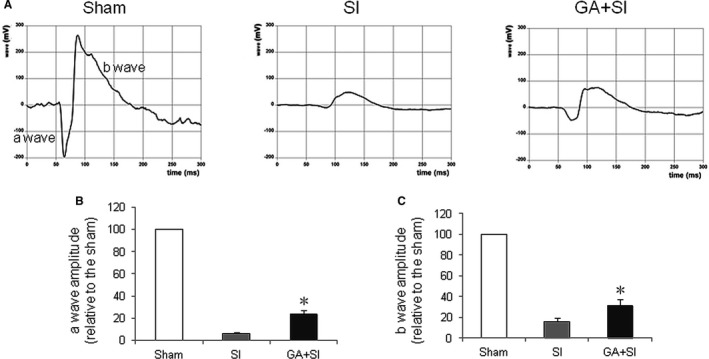
Effects of glycyrrhizin on retinal function after sodium iodate (SI) treatment in vivo. A, Electroretinography measurement of retinal function. a, sham; b, retina treated with SI; c, retina treated with SI plus glycyrrhizin (SI + GA). B, Glycyrrhizin significantly inhibited the decreasing amplitude of a‐wave treated with SI, compared with SI control (*P < 0.05). C, Glycyrrhizin significantly inhibited the decreasing amplitude of b‐wave treated with SI, compared with SI control (*P < 0.05)

To determine whether glycyrrhizin had an effect on the retinal function after SI treatment, the full‐field ERG response was detected. The results showed that glycyrrhizin significantly increased the amplitude of ‘a‐wave’ that originates from the photoreceptors, compared with SI control (Figure 6A,B). Also glycyrrhizin significantly increased the amplitude of ‘b‐wave’ that originates from the bipolar cells, compared with SI control (Figure 6A,C). This indicated that glycyrrhizin improved retinal function after SI treatment.

### 3.6 Glycyrrhizin increased the phosphorylation of AKT in RPE treated with SI

**Figure 7:**
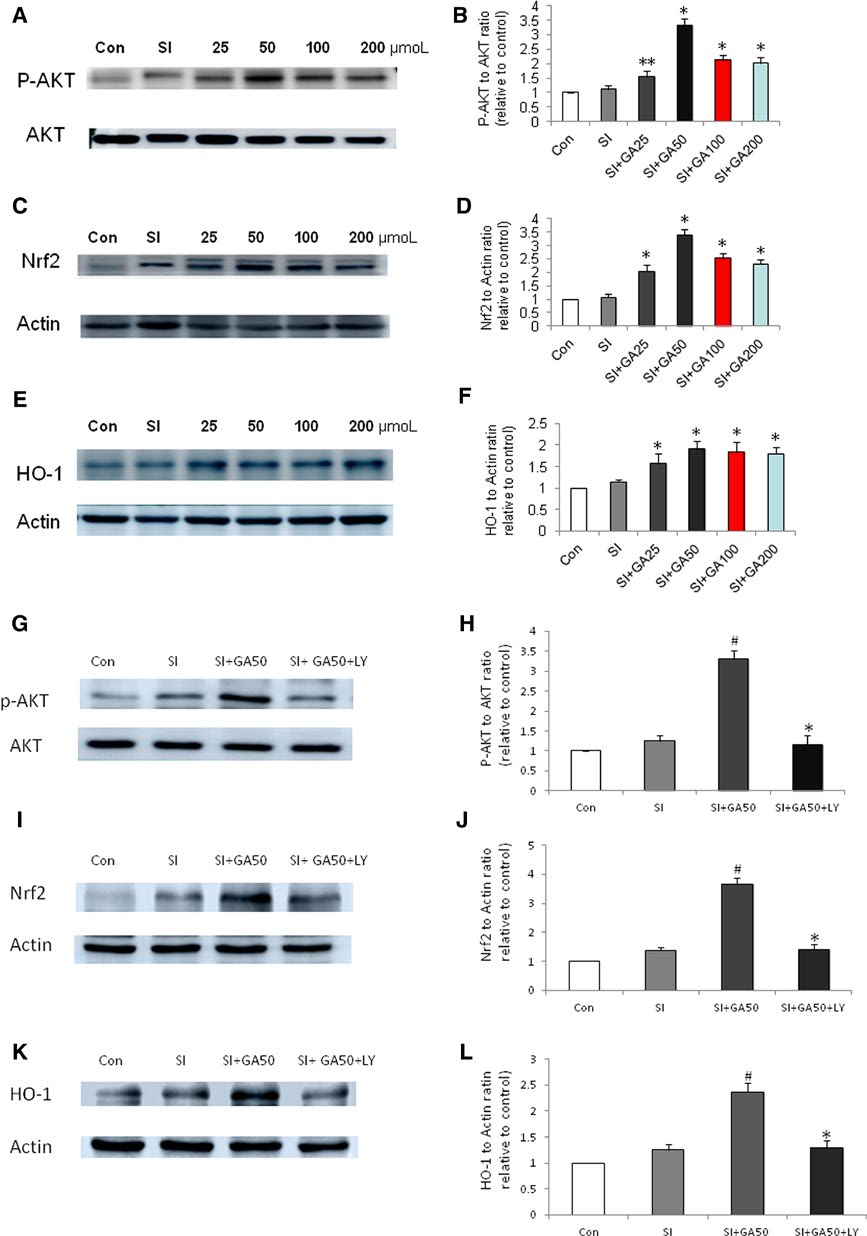
Effects of glycyrrhizin on the expression of phosphorylation of AKT, Nrf2, and HO‐1 in ARPE‐19 cells treated with sodium iodate (SI) by Western blot detection. A, Effects of glycyrrhizin on phosphorylation of AKT. B, Glycyrrhizin significantly increased the phosphorylation of AKT at a concentrations of 25‐200 μmol (**P < 0.05 vs the SI control, *P < 0.01 vs the SI control). C, Effects of glycyrrhizin on the expression of Nrf2. D, Glycyrrhizin significantly increased the expression of Nrf2 at concentrations of 25‐200 μmol (*P < 0.01 vs the SI control). E, Effects of glycyrrhizin on the expression of HO‐1. F, Glycyrrhizin significantly increased the expression of HO‐1 at concentrations of 25‐200 μmol (*P < 0.01 vs the SI control). G, Effects of PI3K/AKT inhibitor LY294002 on phosphorylation of AKT treated with glycyrrhizin. H, Glycyrrhizin significantly increased the phosphorylation of AKT at a concentrations of 50 μmol (^#^ *P* < 0.01 vs the SI control), while LY294002 significantly decreased the phosphorylation of AKT (*P < 0.01 vs the SI + GA50 control). I, Effects of LY294002 on the expression of Nrf2. J, Glycyrrhizin significantly increased the expression of Nrf2 at concentrations of 50 μmol (^#^ *P* < 0.01 vs the SI control), while LY294002 significantly decreased the expression of Nrf2 (*P < 0.01 vs the SI + GA50 control). K, Effects of glycyrrhizin on the expression of HO‐1. L, Glycyrrhizin significantly increased the expression of HO‐1 at concentrations of 50 μmol (^#^ *P* < 0.01 vs the SI control), while LY294002 significantly decreased the expression of HO‐1(*P < 0.01 vs the SI + GA50 control)

Akt pathways play a major role in anti‐apoptosis. Western blot was performed to detect Akt in ARPE‐19 cells treated with SI at 24 hours. Results revealed that glycyrrhizin significantly increased the phosphorylation of Akt in RPE treated with SI (Figure 7A,B), indicating that Akt plays a crucial role in the anti‐apoptosis of RPE by glycyrrhizin after SI treatment.

### 3.7 Glycyrrhizin increased the expression of NRf2 and HO‐1 in the RPE treated with SI

Nrf2/HO‐1 pathways play a major role in anti‐ROS and apoptosis. Western blot was performed to detect the expression of Nrf2 and HO‐1 in ARPE‐19 cells treated with SI at 24 hours. We found that glycyrrhizin significantly increased the expression of Nrf2 and HO‐1 in RPE treated with SI (Figure 7C,F). This suggested that Nrf2/HO‐1 pathway plays an important role in the anti‐ROS and apoptosis of RPE by glycyrrhizin after SI treatment. To further prove that the protective effect of glycyrrhizin on RPE treated with SI is due to the activation of AKT/Nrf2 pathway, the PI3K/AKT inhibitor LY294002 was used concurrently with the SI plus glycyrrhizin. The result showed that LY294002 reversed the effects of glycyrrhizin on the expression of p‐AKT, Nrf2 and HO‐1(Figure 7G‐L).

## 4 DISCUSSION

In this study, we found that glycyrrhizin inhibited SI‐induced ROS in RPE, and decreased RPE cell apoptosis in vitro and retinal cell apoptosis in vivo. To the best of our knowledge, this is the first study that reported glycyrrhizin in the prevention of SI‐induced RPE and retinal toxicity.

Although the mechanisms of toxicity of the eye by SI are not fully elucidated, inducing ROS and subsequently causing retinal apoptosis is one of the effects of SI on the eye. Thus, the strategy against the induction of ROS and apoptosis remained reasonable for the prevention of eye toxicity caused by SI. Several studies have showed that glycyrrhizin inhibited ROS‐mediated photodamage in UV‐B irradiated human skin fibroblasts,^18^ and attenuated alcoholic acid‐induced live injury.^19^ This phenomenon suggested that glycyrrhizin might also reduce SI‐induced eye injury. In this study, we found that glycyrrhizin significantly inhibited ROS in RPE treated with SI, and decreased SI‐induced apoptosis of RPE in vitro and retinal cell apoptosis in vivo. We further confirmed that the thinning of retina was inhibited, the number of drusen was decreased and the retinal function was improved by glycyrrhizin. These indicated that glycyrrhizin prevented against SI‐induced RPE and retinal injury through anti‐ROS and apoptosis.

AKT plays an important role in cell apoptosis.^20^ Numerous studies have showed that phosphorylation of AKT activated cell survival pathways, which in turn prevented the cells from apoptosis.^21^, ^22^, ^23^, ^24^, ^25^ In this study, we found that glycyrrhizin significantly increased phosphorylation of AKT in RPE treated with SI, and significantly decreased apoptosis of RPE. This was in accordance with the studies that demonstrated glycyrrhizin‐mediated protection in H9c2 cells^26^ and neural cells^27^ by increasing the phosphorylation of Akt.

Nrf2 is a crucial regulator of oxidative stress by the activation of endogenous antioxidant enzymes.^28^, ^29^ Normally, Nrf2 is bound to negative regulator Keap1 and is oriented toward the cytoplasm. In the oxidative stress state, Nrf2 dissociates from Keap1 and translocates into the nucleus, inducing the expression of HO‐1 gene that acts as an antioxidative modulator.^30^ Nrf2 protects against cell apoptosis by inhibiting MARKs and NF‐κB, while HO‐1 protects against apoptosis directly or by inhibiting ROS.^31^ Our study demonstrated that glycyrrhizin increased the expression of Nrf2 and HO‐1 and decreased ROS in RPE, and PI3K/AKT inhibitor LY294002 reversed the effect of glycyrrhizin on the pAKT, Nrf2 and HO‐1 in RPE treated with SI. This suggested that the Nrf2/HO‐1 signalling pathway participated in the protection of glycyrrhizin against SI‐induced RPE and retinal apoptosis.

In summary, we found that glycyrrhizin preserves SI‐induced RPE and retinal injury by inhibiting ROS and decreasing apoptosis. The underlying mechanisms include the activation of Akt and Nrf2/HO‐1 pathway by glycyrrhizin, thereby preventing the retinal cells against the SI‐induced toxicity. These results suggested that glycyrrhizin might serve as a putative tool for preventing atrophic AMD in clinical practice.

## CONFLICT OF INTEREST

The authors declare no conflict of interest.

## References

[1] Smith W , Assink J , Klein R , et al. Risk factors for age‐related macular degeneration: pooled findings from three continents. Ophthalmology. 2001;108:697‐704. 11297486 10.1016/s0161-6420(00)00580-7

[2] Friedman DS , O’Colmain BJ , Munoz B , et al. Prevalence of age‐related macular degeneration in the United States. Arch Ophthalmol. 2004;122:564‐572. 15078675 10.1001/archopht.122.4.564

[3] Jager RD , Mieler WF , Miller JW . Age‐related macular degeneration. N Engl J Med. 2008;358:2606‐2617. 18550876 10.1056/NEJMra0801537

[4] Bressler NM , Bressler SB , Fine SL . Age‐related macular degeneration. Surv Ophthalmol. 1988;32:375‐413. 2457955 10.1016/0039-6257(88)90052-5

[5] Bressler NM , Bressler SB , Seddon JM , Gragoudas ES , Jacobson LP . Drusen characteristics in patients with exudative versus non‐exudative age‐related macular degeneration. Retina. 1988;8:109‐114. 3420311 10.1097/00006982-198808020-00005

[6] Ambati J , Fowler BJ . Mechanisms of age‐related macular degeneration. Neuron. 2012;75:26‐39. 22794258 10.1016/j.neuron.2012.06.018 PMC3404137

[7] Narayanan R , Kuppermann BD . Hot topics in dry AMD. Curr Pharm Des. 2017;23:542‐546. 28003009 10.2174/1381612822666161221154424

[8] Seo DY , Lee SR , Heo JW , et al. Ursolic acid in health and disease. Korean J Physiol Pharmacol. 2018;22:235‐248. 29719446 10.4196/kjpp.2018.22.3.235 PMC5928337

[9] Sun X , Zeng H , Wang Q , et al. Glycyrrhizin ameliorates inflammatory pain by inhibiting microglial activation‐mediated inflammatory response via blockage of the HMGB1‐TLR4‐NF‐kB pathway. Exp Cell Res. 2018;369:112‐119. 29763588 10.1016/j.yexcr.2018.05.012

[10] Iino S , Tango T , Matsushima T , et al. Therapeutic effects of stronger neo‐minophagen C at different doses on chronic hepatitis and liver cirrhosis. Hepatol Res. 2001;19:31‐40. 11137478 10.1016/s1386-6346(00)00079-6

[11] vanRossum TG , Vulto AG , de Man RA , Brouwer JT , Schalm SW . Review article: glycyrrhizin as a potential treatment for chronic hepatitis C. Aliment Pharmacol Ther. 1998;12:199‐205. 9570253 10.1046/j.1365-2036.1998.00309.x

[12] Gong G , Xiang L , Yuan L , et al. Protective effect of glycyrrhizin, a direct HMGB1 inhibitor, on focal cerebralischemia/reperfusion‐induced inflammation, oxidative stress, and apoptosis in rats. PLoS ONE. 2014;9:e89450. 24594628 10.1371/journal.pone.0089450 PMC3942385

[13] Franco LM , Zulliger R , Wolf‐Schnurrbusch UE , et al. Decreased visual function after patchy loss of retinal pigment epithelium induced by low‐dose sodium iodate. Invest Ophthalmol Vis Sci. 2009;50:4004‐4010. 19339739 10.1167/iovs.08-2898

[14] Ringvold A , Olsen EG , Flage T . Transient breakdown of the retinal pigment epithelium diffusion barrier after sodium iodate: a fluorescein angiographic and morphological study in the rabbit. Exp Eye Res. 1981;33:361‐369. 7297617 10.1016/s0014-4835(81)80088-7

[15] Kiuchi K , Yoshizawa K , Shikata N , Moriguchi K , Tsubura A . Morphologic characteristics of retinal degeneration induced by sodium iodate in mice. Curr Eye Res. 2002;25:373‐379. 12789545 10.1076/ceyr.25.6.373.14227

[16] Redfern WS , Storey S , Tse K , et al. Evaluation of a convenient method of assessing rodent visual function in safety pharmacology studies: effects of sodium iodate on visual acuity and retinal morphology in albino and pigmented rats and mice. J Pharmacol Toxicol Methods. 2011;63:102‐114. 20619348 10.1016/j.vascn.2010.06.008

[17] Enzmann V , Row BW , Yamauchi Y , et al. Behavioral and anatomical abnormalities in a sodium iodate‐induced model of retinal pigment epithelium degeneration. Exp Eye Res. 2006;82:441‐448. 16171805 10.1016/j.exer.2005.08.002

[18] Farrukh MR , Nissar UA , Kaiser PJ , et al. Glycyrrhizic acid (GA) inhibits reactive oxygen Species mediated photodamage by blocking ER stress and MAPK pathway in UV‐B irradiated human skin fibroblasts. J Photochem Photobiol B. 2015;148:351‐357. 26009870 10.1016/j.jphotobiol.2015.05.003

[19] Huo X , Yang S , Sun X , Meng X , Zhao Y . Protective effect of glycyrrhizic acid on alcoholic liver injury in rats by modulating lipid metabolism. Molecules. 2018;23 10.3390/molecules23071623. PMC6100631 29973492

[20] Datta SR , Dudek H , Tao Y , et al. Akt phosphorylation of BAD couples survival signals to the cell‐intrinsic death machinery. Cell. 1997;91:231‐241. 9346240 10.1016/s0092-8674(00)80405-5

[21] Huang J , Inoue M , Hasegawa M , et al. Sendai viral vector mediated angiopoietin‐1 gene transfer for experimental ischemic limb disease. Angiogenesis. 2009;12:243‐249. 19322669 10.1007/s10456-009-9144-6

[22] Piao W , Wang H , Inoue M , Hasegawa M , Hamada H , Huang J . Transplantation of Sendai viral angiopoietin‐1‐modified mesenchymal stem cells for ischemic limb disease. Angiogenesis. 2010;13:203‐210. 20458615 10.1007/s10456-010-9169-x

[23] Matsui T , Nagoshi T , Rosenzweig A . Akt and PI3‐kinase signaling in cardiomyocyte hypertrophy and survival. Cell Cycle. 2003;2:220‐223. 12734428

[24] Sugden PH . Ras, Akt, and mechanotransduction in the cardiac myocyte. Circ Res. 2003;93(12):1179‐1192. 14670833 10.1161/01.RES.0000106132.04301.F5

[25] Wang YK , Deng F , Miao J , et al. Neuroprotection by carbenoxolone against ischemia injury involves PI3K/Akt pathway. Clin Lab. 2015;61:1561‐1568. 26642720 10.7754/clin.lab.2015.150215

[26] Wang L , Zhang Y , Wan H , et al. Glycyrrhetinic acid protects H9c2 cells from oxygen glucose deprivation‐induced injury through the PI3K/AKT signaling pathway. J Nat Med. 2017;71:27‐35. 27406329 10.1007/s11418-016-1023-z

[27] Teng L , Kou C , Lu C , et al. Involvement of the ERK pathway in the protective effects of glycyrrhizic acid against the MPP+-induced apoptosis of dopaminergic neuronal cells. Int J Mol Med. 2014;34:742‐748. 24993693 10.3892/ijmm.2014.1830 PMC4121344

[28] Kwak MK , Wakabayashi N , Kensler TW . Chemoprevention through the Keap1‐Nrf2 signaling pathway by phase 2 enzyme inducers. Mutat Res. 2004;555:133‐148. 15476857 10.1016/j.mrfmmm.2004.06.041

[29] Kensler TW , Wakabayashi N , Biswal S . Cell survival responses to environmental stresses via the Keap1‐Nrf2‐ARE pathway. Annu Rev Pharmacol Toxicol. 2007;47:89‐116. 16968214 10.1146/annurev.pharmtox.46.120604.141046

[30] Baird L , Llères D , Swift S , Dinkova‐Kostova AT . Regulatory flexibility in the Nrf2‐mediated stress response is conferred by conformational cycling of the Keap1‐Nrf2 protein complex. Proc Natl Acad Sci USA. 2013;110:15259‐15264. 23986495 10.1073/pnas.1305687110 PMC3780858

[31] So HS , Kim HJ , Lee JH , et al. Flunarizine induces Nrf2‐mediated transcriptional activation of heme oxygenase‐1 in protection of auditory cells from cisplatin. Cell Death Differ. 2006;13:1763‐1775. 16485034 10.1038/sj.cdd.4401863

